# Prognostic relevance of a T-type calcium channels gene signature in solid tumours: A correlation ready for clinical validation

**DOI:** 10.1371/journal.pone.0182818

**Published:** 2017-08-28

**Authors:** Lorenzo Fornaro, Caterina Vivaldi, Dong Lin, Hui Xue, Alfredo Falcone, Yuzhuo Wang, Francesco Crea, Martin D. Bootman

**Affiliations:** 1 Unit of Medical Oncology 2, Azienda Ospedaliero-Universitaria Pisana, Pisa, Italy; 2 Experimental Therapeutics, BC Cancer Research Centre, Vancouver, British Columbia, Canada; 3 School of Life, Health & Chemical Sciences, The Open University, Walton Hall, Milton Keynes, United Kingdom; Sudbury Regional Hospital, CANADA

## Abstract

**Background:**

T-type calcium channels (TTCCs) mediate calcium influx across the cell membrane. TTCCs regulate numerous physiological processes including cardiac pacemaking and neuronal activity. In addition, they have been implicated in the proliferation, migration and differentiation of tumour tissues. Although the signalling events downstream of TTCC-mediated calcium influx are not fully elucidated, it is clear that variations in the expression of TTCCs promote tumour formation and hinder response to treatment.

**Methods:**

We examined the expression of TTCC genes (all three subtypes; *CACNA-1G*, *CACNA-1H* and *CACNA-1I*) and their prognostic value in three major solid tumours (*i*.*e*. gastric, lung and ovarian cancers) via a publicly accessible database.

**Results:**

In gastric cancer, expression of all the *CACNA* genes was associated with overall survival (OS) among stage I-IV patients (all p<0.05). By combining the three potential biomarkers, a TTCC signature was developed, which retained a significant association with OS both in stage IV and stage I-III patients. In lung and ovarian cancer, association with OS was also significant when all tumour stages were considered, but was partly lost or inconclusive after splitting cases into localized and metastatic subsets.

**Conclusions:**

Alterations in *CACNA* gene expression are linked to tumour prognosis. Gastric cancer represents the most promising setting for further evaluation.

## Introduction

Calcium is a universal cellular messenger that regulates a plethora of physiological events including gene transcription, cell survival and cell proliferation [1,2]. Numerous studies have shown that cellular calcium signalling is altered in cancer cells, although not all tumour types show the same alterations in calcium signalling [3]. Changes in calcium signals affect cell proliferation, apoptosis and metastatic potential [4].

Among the different calcium channels implicated in cancer, T-type calcium channels (TTCCs) have been linked to tumour initiation and progression [3]. In particular, TTCCs trigger cancer cell proliferation via the mitogen-activated kinase pathway [5], while TTCC inhibition induces cancer cell apoptosis [4]. Pharmacological TTCC inhibitors are being tested as anti-cancer drugs in clinical trials [6]. Therefore, modulating calcium homeostasis via inhibition of TTCCs may be a promising strategy for cancer treatment, and this approach is under early phase of development in several tumour types [7].

TTCCs consist of a calcium-conducting alpha subunit that forms a complex with a number of other regulatory subunits. Three types of TTCC-alpha subunits have been identified (named Cav3.1, Cav3.2 and Cav3.3), encoded by the genes *CACNA-1G*, *CACNA-1H* and *CACNA-1I* [8]. Expression of the three *CACNA* genes varies in different tumour types, and between normal tissue and the corresponding neoplastic counterpart [4]. In particular, no study has investigated how variations in TTCC expression impact on patient prognosis, or response to conventional treatments. Given the burgeoning evidence that suggests a causal link between TTCC expression and tumour progression, it is critical to examine the utility of TTCCs as a prognostic and therapeutic tool.

In order to explore the role of *CACNA-1G*, *CACNA-1H* and *CACNA-1I* in the clinical scenario, we searched a publicly available database [9] and evaluated the association between the expression of these genes and survival in different solid tumours represented on the online platform (*i*.*e*. breast, gastric, lung and ovarian cancer). These tumours represent unmet needs in clinical oncology, due to the inadequate therapeutic alternatives currently available for the majority of patients, particularly in the advanced disease setting. Moreover, many of the attempts made in the last years to improve the results of conventional chemotherapy by means of molecularly targeted drugs have disappointingly failed, as in the case of gastric cancer [10]. Therefore, the identification of new promising prognostic biomarkers, which may also represent specific and potent therapeutic targets, is a critical demand in oncology.

## Materials and methods

We analyzed data from the world’s largest collection of patients’ transcriptomic data for breast, gastric, lung and ovarian cancer (Kaplan-Meier Plotter, accessible on: http://kmplot.com/analysis/) [9]. We queried the database for expression of three TTCC-encoding genes: *CACNA-1G*, *CACNA-1H* and *CACNA-1I*. For all tumour types, association between gene expression and survival parameters available on the online platform was investigated. Survival parameters used were the following: overall survival (OS) and survival without tumour progression for advanced (M1) cases (progression-free survival, PFS) or tumour recurrence for non-metastatic (M0) cases (disease-free survival, DFS).

A log-rank test was used to correlate survival outcomes among subgroups identified by gene expression, and the effect was estimated by means of hazard ratio (HR) and corresponding 95% confidence interval (95%CI). The level of significance for single gene analyses was set at p<0.05. Association with OS in the entire dataset of patients for each tumour types was initially tested. Cases were then categorized according to the most relevant prognostic variable (*i*.*e*. tumour stage according to TNM classification [accessible on https://cancerstaging.org/Pages/default.aspx]), separating metastatic cases (stage IV, M1) and potentially curable disease (stage I-III, M0). For each single tumour type, other subgrouping of available data beyond disease stage was applied as appropriate, exploiting the variables available on the database. In detail, for lung cancer, histology (adenocarcinoma *versus* squamous-cell carcinoma) was used, whereas for ovarian cancer the quality of resection (optimal debulking surgery *versus* suboptimal debulking surgery) was selected. For breast cancer, different covariates were looked for to adjust results and allow appropriate interpretation of the results (HER-2 status, hormone receptors status, tumour grading, treatment received). However, as the database does not allow sufficient patient stratification according to established prognostic and predictive parameters and information about the treatment administered is limited, we decided not to explore the role of *CACNA* genes expression in breast cancer.

When results across the subgroups were found to be consistent, step-down correction was applied to adjust for multiple tests. All the genes significantly associated with clinical outcome after adjustment were combined to generate a TTCC gene signature, whose prognostic significance was investigated across subgroups by means of the log-rank test and setting significance at p<0.05.

In order to further elucidate the role of *CACNA* genes in solid tumours, the online database of the Living Tumor Lab (Vancouver, BC, Canada) was queried (accessible on: http://www.livingtumorlab.com/): the levels of *CACNA* expression in lung and ovarian cancer xenografts from patient-derived samples was investigated (gastric cancer was not evaluable on the platform, as no xenografts from gastric cancer patients has been developed yet). Patient-derived sub-renal capsule xenografts were generated as described in previous publications [11,12]. Transcriptomic analyses, expressed as log base-2 (log2) microarray data, from these xenografts were generated as described in the original paper [13] and are deposited at the Living Tumor Lab website. The expression values for each gene were reported on a transformed log2 scale. A Mann-Whitney test was used to compare different *CACNA* gene expression between metastatic and non-metastatic xenografts.

## Results

### Gastric cancer

*CACNA-1H* was the single best predictor of clinical outcome (Table 1), showing significant association with OS in both stage I-III (HR 2.27, 95%CI 1.62–3.20; p<0.001) and stage IV (HR 1.8, 95%CI 1.22–2.67; p = 0.003) patients, with high expression associated with poorer survival outcomes. When tested for association with DFS and PFS, *CACNA-1H* retained its prognostic value (HR 2.03, 95%CI 1.47–2.80; p<0.001 and HR 1.62, 95%CI 1.10–2.39; p = 0.013, respectively).

**Table 1:** Association of CACNA-1G, CACNA-1H and CACNA-1I with outcome in gastric cancer.

Gene	*n*	Stage	DFS/PFS			OS		
	FP/OS		HR	95%CI	p	HR	95%CI	p
*CACNA1G*	-/876	all	not applicable			0.84	0.71–1.00	0.046
	443/444	I-III, M0	0.84	0.63-1-13	0.250	0.85	0.62–1.15	0.290
	141/148	IV	0.66	0.43–1.03	0.063	1.57	1.01–2.45	0.044
*CACNA1H*	-/876	all	not applicable			1.64	1.38–1.94	<0.001
	443/444	I-III, M0	2.03	1.47–2.80	<0.001	2.27	1.62–3.20	<0.001
	141/148	IV	1.62	1.10–2.39	0.013	1.80	1.22–2.67	0.003
*CACNA1I*	-/876	all	not applicable			1.53	1.28–1.82	<0.001
	443/444	I-III, M0	1.59	1.17–2.15	0.003	1.60	1.19–2.14	0.002
	141/148	IV	1.34	0.92–1.97	0.130	1.60	1.09–2.35	0.015

**Abbreviations:** *n*, number of patients (for first progression [FP] analysis–*i*.*e*. DFS and PFS—and OS analysis); DFS, disease-free survival; PFS, progression-free survival; OS, overall survival; HR, hazard ratio; 95%CI, 95% confidence interval; p, p-value.

Expression of both *CACNA-1G* and *CACNA-1I* was associated with OS in all stage gastric cancer patients, but with opposite effects in terms of survival (lower risk for higher CACNA-1G expression, increased risk for higher *CACNA-1I* expression: HR 0.84, 95%CI 0.71–1.00; p = 0.046 and HR 1.53, 1.28–1.82; p<0.001, respectively), even though the relationship with outcome was less clear and unequivocal among different stages or in terms of DFS and PFS (Table 1).

Moving from the results observed in stage IV disease, we selected the correlations that retained statistical significance by step-down correction for multiple testing. Then, we generated a TTCC gene signature by combining all the three significant *loci* together and calculating the value of (CACNA-1H*CACNA-1I)/CACNA-1G for each patient included. Table 2 shows the results of this analysis: our signature was highly significantly associated with OS, PFS and DFS, in both metastatic and non-metastatic patients (all p<0.05).

**Table 2:** Association of TTCC gene signature with outcome in gastric cancer.

Stage	*n*	DFS/PFS			OS		
	FP/OS	HR	95%CI	p	HR	95%CI	p
all	-/444	not applicable			1.75	1.47–2.09	<0.001
I-III, M0	443/444	2.00	1.43–2.80	<0.001	1.90	1.43–2.51	<0.001
IV	141/148	1.75	1.10–2.79	0.018	1.88	1.21–2.92	0.005

**Abbreviations:** *n*, number of patients (for first progression [FP] analysis–*i*.*e*. DFS and PFS—and OS analysis); DFS, disease-free survival; PFS, progression-free survival; OS, overall survival; HR, hazard ratio; 95%CI, 95% confidence interval; p, p-value.

**Fig 1:**
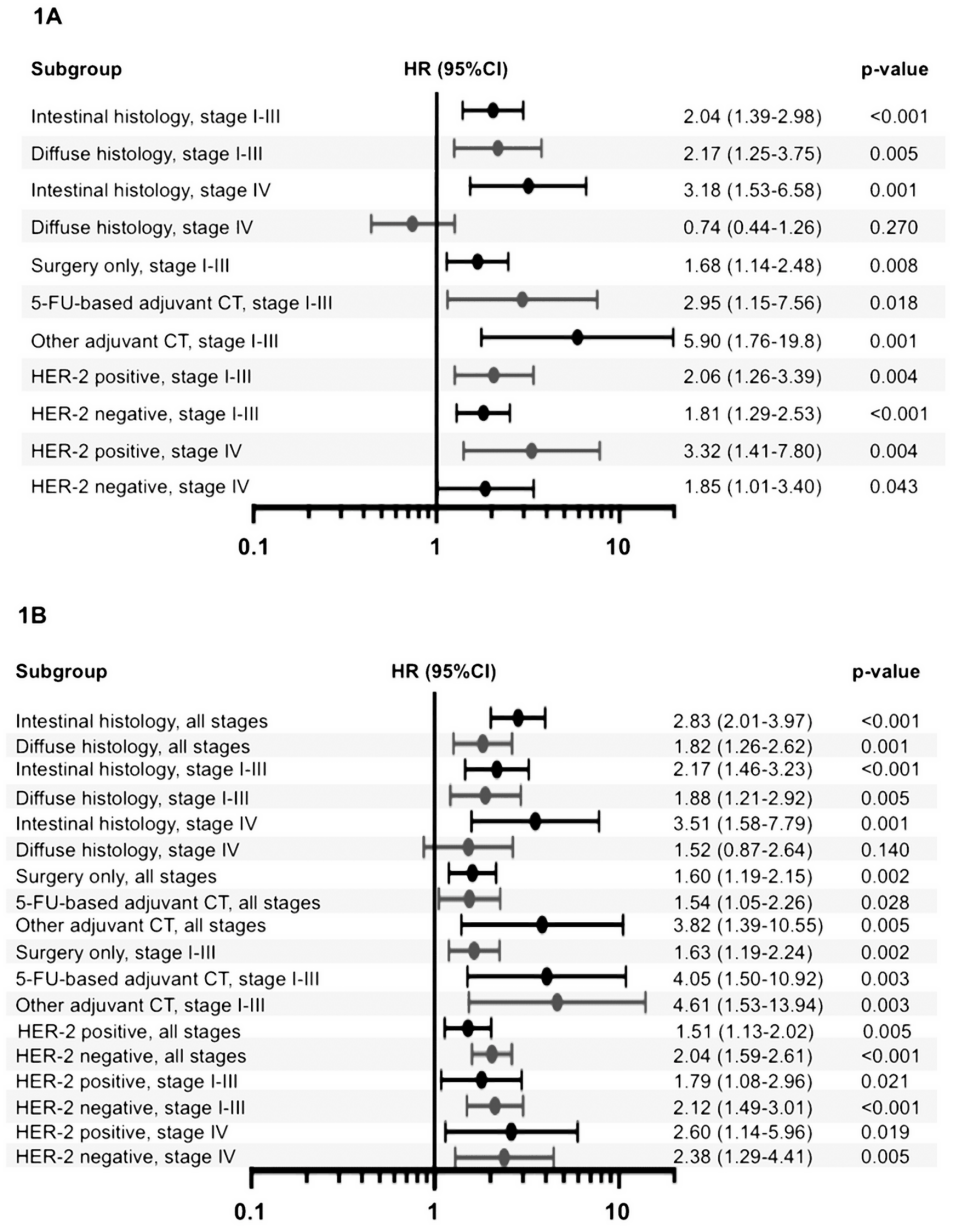
Association of the TTCC gene signature with outcome in gastric cancer: Subgroup analyses for first progression (Fig 1A) and overall survival (Fig 1B). **Abbreviations:** HR (95%CI), hazard ratio (95% confidence interval); p, p-value; 5-FU, 5-fluorouracil; CT, chemotherapy.

In order to refine the role of the TTCC gene signature in gastric cancer, we also tested the prognostic value of our signature in subgroups defined by: Lauren histotypes (intestinal, diffuse), adjuvant treatment (surgery only, 5-fluorouracil-based adjuvant chemotherapy, other chemotherapy) and HER-2 status (positive, negative). Subgroup analyses confirmed the role of the developed gene signature in this setting, demonstrating that it could represent an independent predictor of DFS/PFS (Fig 1A) and OS (Fig 1B).

### Lung and ovarian cancer

Results for other malignancies are summarised in **Supporting Information**. With regard to lung cancer (S1 Table), we queried *CACNA-1G*, *CACNA-1H* and *CACNA-1I* expression in adenocarcinomas and squamous-cell carcinomas (operable and metastastic tumours. With regard to OS, we observed an association similar to that reported in gastric cancer (increased risk for higher *CACNA-1H* and *CACNA-1I* expression, reduced risk for higher *CACNA-1G* expression) in the adenocarcinoma subset when all patients were included, whereas significance was retained for *CACNA-1H* only in squamous-cell carcinoma. We attempted to explore the role of these *loci* in stage I-III versus stage IV patients, but the results were less reliable due to the limited subgroup size. In particular, among stage I-III patients only few cases had received a radical (R0) tumour resection.

When ovarian cancer cases were considered, we observed an opposite trend for all CACNA *loci* compared to gastric and lung adenocarcinoma (S2 Table): when all disease stages were analyzed, higher expression of *CACNA-1G* seemed to increase the risk of death (HR 1.22, 95%CI 1.07–1.40; p = 0.004), whereas higher expression of *CACNA-1H* and *CACNA-1I* reduced the risk (HR 0.82, 95%CI 0.71–0.96; p = 0.011 and HR 0.84, 0.73–0.97; p = 0.018, respectively). Globally considered, however, the effect seemed weaker compared to that observed in gastric cancer. As quality of surgery is a key element in the prognosis of all ovarian cancer cases, particularly those with more advanced disease, we analyzed earlier stages (I-II) separately to advanced ones (stage III-IV), restricting the analysis to stage III-IV patients with optimal debulking surgery: as reported in S2 Table, the association with outcome was retained in some comparisons, but none of the investigated genes retained its significant effect on all survival endpoints (DFS/PFS and OS). Results did not change significantly when only patients treated with platinum-containing chemotherapy were included in the analyses (S2 Table).

### Transcriptomic analysis of CACNA-dependent pathways

**Fig 2:**
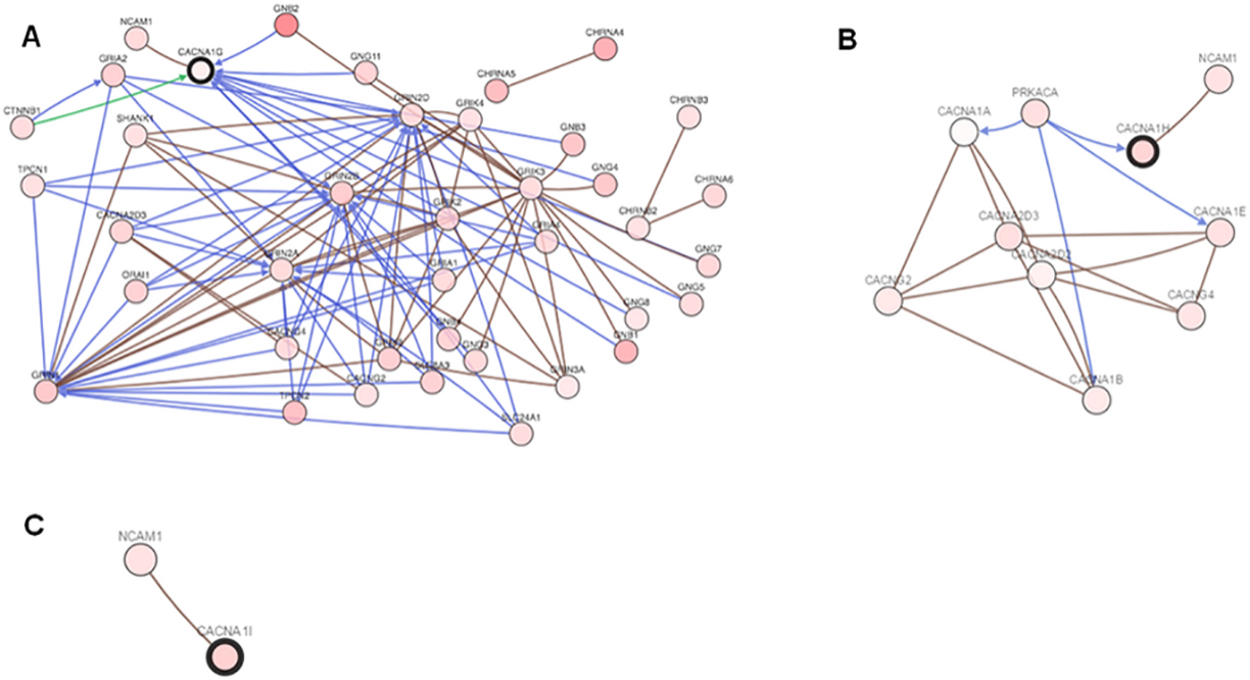
Network analyses for CACNA1G (A), CANA1H (B) and CACNA1I (C). Red color is more intense when associated with higher percentage of up-regulation in cancer samples. Blue lines mean: “controls the state change of”; green lines mean: “controls the expression of”; brown lines mean: “in complex with”.

To gain further insight into the molecular mechanisms underpinning the prognostic role of *CACNA* genes, we performed a molecular network analysis, using the Cbio portal (stomach adenocarcinoma database, 265 clinical samples) [14]. *CACNA-1G* was up-regulated in 25% of these clinical samples (Z score>0), and it was associated with 51 interacting molecules (Fig 2A). In particular, it was predicted to be regulated by beta-catenin (CTNNB1) and in complex with neural adhesion molecule (NCAM1). *CACNA-1I* (up-regulated in 25% of samples) was part of a 29-node network, in association with NCAM1 (Fig 2B). *CACNA-1H* was also predicted to be modulated by PRKACA (Protein Kinase cAMP-Activated Catalytic Subunit Alpha). The only prediction available for *CACNA-1I* (upregulated in 27% of samples) was the association with NCAM1 (Fig 2C).

### *CACNA* genes expression in patient-derived cancer tissue xenografts

In order to confirm the expression of *CACNA* genes in an independent dataset, we analysed transcriptomic data from high-fidelity patient-derived xenografts. Results for lung and ovarian cancer are summarized in S1 and S2 Figs, respectively. With regard to lung cancer, no apparent relevant differences among the different *CACNA loci* was evident, with the exception of *CACNGA-1H* which was highly expressed in two samples (L347 and L613), differing for histology (adenocarcinoma and squamous-cell carcinoma, respectively) and clinical stage (stage IV and IIIA respectively). When metastatic xenografts were compared with non-metastatic ones, higher *CACNA-1I* expression was evident in the first subset (p = 0.022) (S3 Fig). Among the available ovarian cancer xenografts, no gross imbalances in the expression of candidate genes were reported. The only differences were observed for *CACNA-1H* (whose expression was higher in two samples: LTL-175 and LTL-307) and *CACNA-1I* (which appeared to be more expressed in two samples: LTL-237 and LTL-273). However, when metastatic and non-metastatic ovarian cancer xenografts were compared, no difference in expression was reported for any *CACNA* gene evaluated (all p>0.05).

## Discussion

TTCCs were identified in the 1980s by Tsien and colleagues, and have tissue expression patterns and biophysical characteristics that distinguish them from other voltage-operated Ca^2+^ channels [15,16]. In particular, they can be activated by relatively small membrane depolarisations. Hence, the term ‘low voltage activated channels’ is often used to describe them. The functional consequence of this characteristic is that the voltage range for activation overlaps with that for inactivation, which is close to the resting membrane of most cells, giving the potential for sub-populations of TTCCs to be perpetually active and drive a constitutive Ca^2+^ influx current that is sometimes referred to as a ‘window current’ [17].

TTCCs have been proposed to be involved in action potential bursting in neurons, endocrine cell secretion, pacemaking activity in the heart, as well as in pathological conditions such as epilepsy and autism [18]. Moreover, numerous reports have correlated the expression of TTCCs with proliferation of tumour cells [19,20,21,22].

Our analyses suggest for the first time that a TTCC gene signature accurately predicts the prognosis of gastric cancer patients, both in the radically resectable and metastatic disease. Moreover, some of these TTCC-related genes may play a role in ovarian cancer, although the overall magnitude of effect seems lower compared to gastric cancer. Taken together, these data confirm that monitoring *CACNA-1G*, *CACNA-1H* and *CACNA-1I* expression in solid tumours may be an intriguing approach to refine patient stratification and hopefully identified new therapeutic targets. As the identification of innovative prognostic and predictive biomarkers is a major need in modern oncology, the preliminary evidence of a putative impact of *CACNA* genes expression on patient survival both in the advanced and in the potentially curative settings prompts further research on the biological mechanisms behind TTCCs functions in solid tumours. Indeed, accurately predicting patient prognosis allows for a more rationale study design and treatment allocation, as well as identifying new potential targets for anticancer treatment open the way to hypothesize a personalized approach in the development of calcium channels inhibitors.

In the gastric cancer subset of a large, public database of transcriptomic data the gene signature described by (CACNA-1H*CACNA-1I)/CACNA-1G was associated with both OS and DFS/PFS, even in subgroups defined by tumour stage, HER-2 expression, type of adjuvant chemotherapy and Lauren histology. These findings deserve further confirmation in larger, prospectively collected series treated with homogenous local and systemic treatments. Our analysis shares the limitations of any retrospective investigation, potentially biased by different confounding factors. Moreover, the platform did not allow for appropriate patient stratification by quality of surgery in resected cases and type of chemotherapy among metastatic patients. Therefore, we cannot exclude a potential impact of other known determinants of prognosis beyond TTCC genes. However, the concordance of the findings in several subgroups defined by the covariates available on KMPlot prompts in our opinion the evaluation of this putative biomarker in this specific tumour type.

With regard to other malignancies available for analyses, some considerations need to be kept in mind in the interpretation of the results. For ovarian cancer, *CACNA-1G*, *CACNA-1H* and *CACNA-1I* all showed a significant association with OS when all the cases were included in the analysis, but the direction of the effect was exactly the opposite of that shown in gastric cancer. For some of the comparisons, significance was retained also after adjustment for tumour stage, quality of surgery and type of chemotherapy. Other known prognostic factors are available on the platform (*i*.*e*. grade, histology, CA125 levels), but the number of cases would have been too limited with stricter selection criteria. Data from patient-derived xenografts highlighted some variations in CACNA expression among samples, particularly for *CACNA-1H* and *CACNA-1I*, which are both clinically associated with a reduction in the risk of death according to KMPlot analyses.

TTCC genes also showed some significant association with OS in lung cancer, in particular in the adenocarcinoma subset, and the observed direction of the effect of expression of *CACNA-1G*, *CACNA-1H* and *CACNA-1I* was in the same way to that reported in gastric cancer. However, other factors (mainly related to driver gene alterations in lung cancer, such as *EGFR* and *ALK*) were not reported, as well as the information about treatment with targeted therapy for oncogene-addicted tumours (which may confound the results in the adenocarcinoma subset). Data from the Living Tumor Lab platform revealed that some xenografts showed particularly high *CACNA1H* expression, but no correlation seems to exist between this parameter and clinical (e.g. stage) or pathological (e.g. histology) features.

Our network analysis indicates that the 3 *CACNA* genes are associated with partially overlapping, but distinct molecular networks. Interestingly, all 3 CACNAs were predicted to interact closely with NCAM1, which has been positively associated with gastric cancer progression [23]. Notably, NCAM1 has been shown to trigger intracellular signalling via TTCCs in neurons [24]. It would be interesting to see if NCAM1 plays a similar role in gastric and other neoplasms. CACNA1G was associated with the most complex molecular network, which included several G-proteins and CTNNB1, a proto-oncogene that drives gastric cancer cells’ epithelial-to-mesenchymal transition [25]. We believe that these network analyses shed new light on the specific contribution of each *CACNA* gene to neoplastic progression. Further molecular studies are needed to dissect the specific role of each of these molecular pathways. In particular, studies are needed to establish that calcium signals, arising via functional TTCC activation, are actually mediating the effects of *CACNA* gene expression on neoplastic progression. Since calcium channels often work in macromolecular assemblies within cells, it is possible that there are both calcium-dependent and calcium-independent signalling partners for TTCCs. Moreover, future insights into TTCC-related pathways may pave the way toward a more rationale preclinical and clinical development for agents targeting calcium homeostasis via TTCCs inhibition.

Blocking TTCC activity with an endogenous protein [26], siRNA-based/RNAi knockdown [5,22,27,28,29,30] or pharmacological inhibitors [22,28,30,31,32,33] has been shown to reduce cancer cell proliferation and migration. Furthermore, inhibiting TTCCs enhances the effectiveness of conventional anti-cancer chemotherapeutic agents [4].

Many studies have used pharmacological inhibitors, such as the compounds mibefradil, NNC-55-0396, KYS05090 and pimozide, to investigate the role of TTCCs in tumour cell proliferation. These compounds have been shown to prevent proliferation of various types of tumour cells, with effective concentrations in the low micromolar range [31]. Inhibitors of ‘high voltage activated channels’, such as the L-type Ca^2+^ channel blocker nifedipine, do not share the anti-proliferative potency of TTCC antagonists [28]. Although some studies have indicated that pharmacological TTCC blockers may affect cell proliferation via off-target effects, there is considerable evidence from other approaches that substantiates the involvement of TTCCs in tumour cell growth. For example, RNAi-mediated reduction of TTCC expression was found to inhibit the proliferation of tumour cells in which a *bona fide* T-type Ca^2+^ current was also demonstrated, but not in tumour cells that did not have a T-type Ca^2+^ current [27]. In addition, oral administration of mibefradil to mice bearing gliobastoma xenografts was found to inhibit tumour growth and prolong survival of the host animals [22].

Despite numerous studies showing that inhibiting or downregulating TTCCs affects tumour cell proliferation, the molecular events leading from reduced TTCC activity to inhibition of cell growth are unclear [4]. Indeed, the reported cellular effects of reduced TTCC activity are widely disparate and include cell cycle arrest [32,29], induction of necrosis [31], increased p21^*CIP1*^ expression [27], apoptotic cell death [5,21,32], reduced mTORC2/Akt signalling [22,28], and stimulation of p38 MAP kinase activity [5]. Whilst there is still more to discover before a full understanding of the role of TTCCs in cancer cell proliferation is established, the burgeoning literature on this topic has provoked suggestions that TTCCs blockers designed for other functions could be repurposed for cancer therapy [34]. While waiting for these insights into TTCC-mediated mechanisms of tumour progression, our signature could serve as a promising instrument to drive research into the tailored development of Ca2+ channels-targeting agents and deserves further validarion, at least in gastric cancer.

In conclusion, our results are in line with preclinical data supporting the role of TTCCs in tumour progression and suggest a potential role of TTCC genes in predicting patient outcome in the clinical scenario. On the basis of the investigated platforms, gastric cancer represents a promising setting for the prospective validation of these findings. More studies examining the link between TTCC gene expression and tumour progression are needed, in addition to investigations into the cellular consequences of TTCC expression in tumour cells to develop therapeutic targets.

## Supporting information

S1 Table Association of CACNA-1G, CACNA-1H and CACNA-1I with outcome in lung cancer. **Abbreviations:** *n*, number of patients (for first progression [FP] analysis–*i*.*e*. DFS and PFS—and OS analysis); DFS, disease-free survival; PFS, progression-free survival; OS, overall survival; HR, hazard ratio; 95%CI, 95% confidence interval; p, p-value; *only R0-resected cases included.
(PDF)

S2 Table Association of CACNA-1G, CACNA-1H and CACNA-1I with outcome in ovarian cancer. **Abbreviations:** *n*, number of patients (for first progression [FP] analysis–*i*.*e*. DFS and PFS—and OS analysis); DFS, disease-free survival; PFS, progression-free survival; OS, overall survival; HR, hazard ratio; 95%CI, 95% confidence interval; p, p-value; *only cases with optimal debulking surgery included.
(PDF)

S1 Fig Expression levels of different CACNA (transformed log2) from microarray analysis of patient-derive lung cancer xenografts. **Abbreviations:** NOS, not otherwise specified. TNM stage refers to tumour stage in the original patient: all xenografts were derived from primary lung tumours without distant metastases (M0), with the only exception of L347 (derived from a metastatic lung tumour, M1).
(TIF)

S2 Fig Expression levels of different CACNA (transformed log2) from microarray analysis of patient-derive ovarian cancer xenografts. **Abbreviations:** Histology refers to tumour type in the original patient: all xenografts were derived from primary ovarian tumours.
(TIF)

S3 Fig Expression levels of CACNA1I (transformed log2) from microarray analysis of patient-derive lung cancer xenografts (metastatic *vs*. non-metastatic). (TIF)
