# Supplementary material for: Prognostic relevance of a T-type calcium channels gene signature in solid tumours: A correlation ready for clinical validation
Source: PLoS One. 2017 Aug 28;12(8):e0182818. doi: 10.1371/journal.pone.0182818 (PMC5573204; doi:10.1371/journal.pone.0182818)
Supplement: S1 Table — Abbreviations: n, number of patients (for first progression [FP] analysis–i.e. DFS and PFS—and OS analysis); DFS, disease-free survival; PFS, progression-free survival; OS, overall survival; HR, hazard ratio; 95%CI, 95% confidence interval; p, p-value; *only R0-resected cases included. (PDF) [file pone.0182818.s001.pdf]

Supplementary Table 1. Association of CACNA-1G, CACNA-1H and CACNA-1I with outcome in lung cancer

Lung cancer (adenocarcinoma)								
**Gene**	***n***	**Stage**	**DFS/PFS**			**OS**		
	**FP/OS**		**HR**	**95%CI**	**p**	**HR**	**95%CI**	**p**
CACNA1G	-/720	all	not applicable			0.62	0.49-0.78	<0.001
	0/0	I-III, M0*	not evaluated (no R0 cases)			not evaluated (no cases)		
	0/4	IV	not evaluated (no cases)			not evaluated (number too low)		
CACNA1H	-/720	all	not applicable			1.68	1.31-2.15	<0.001
	0/0	I-III, M0*	not evaluated (no cases)			not evaluated (no cases)		
	0/4	IV	not evaluated (no cases)			not evaluated (number too low)		
CACNA1I	-/720	all	not applicable			1.55	1.19-2.02	0.001
	0/0	I-III, M0*	not evaluated (no cases)			not evaluated (no cases)		
	0/4	IV	not evaluated (number too low)			not evaluated (number too low)		
Lung cancer (squamous-cell carcinoma)								
**Gene**	***n***	**Stage**	**DFS/PFS**			**OS**		
			**HR**	**95%CI**	**p**	**HR**	**95%CI**	**p**
CACNA1G	-/524	all	not applicable			1.20	0.92-1.58	0.185
	0/62	I-III, M0*	not evaluated (no cases)			0.61	0.28-1.32	0.202
	0/0	IV	not evaluated (no cases)			not evaluated (no cases)		
CACNA1H	-/524	all	not applicable			1.35	1.04-1.75	0.024
	0/62	I-III, M0*	not evaluated (no cases)			1.66	0.76-3.63	0.198
	0/0	IV	not evaluated (no cases)			not evaluated (no cases)		
CACNA1I	-/524	all	not applicable			1.17	0.91-1.15	0.233
	0/62	I-III, M0*	not evaluated (no cases)			1.59	0.69-3.69	0.274
	0/0	IV	not evaluated (no cases)			not evaluated (no cases)		

Abbreviations: *n*, number of patients (for first progression [FP] analysis – *i.e.* DFS and PFS – and OS analysis); DFS, disease-free survival; PFS, progression-free survival; OS, overall survival; HR, hazard ratio; 95%CI, 95% confidence interval; p, p-value; *only R0-resected cases included.
