# Supplementary material for: Prognostic relevance of a T-type calcium channels gene signature in solid tumours: A correlation ready for clinical validation
Source: PLoS One. 2017 Aug 28;12(8):e0182818. doi: 10.1371/journal.pone.0182818 (PMC5573204; doi:10.1371/journal.pone.0182818)
Supplement: S2 Table — Abbreviations: n, number of patients (for first progression [FP] analysis–i.e. DFS and PFS—and OS analysis); DFS, disease-free survival; PFS, progression-free survival; OS, overall survival; HR, hazard ratio; 95%CI, 95% confidence interval; p, p-value; *only cases with optimal debulking surgery included. (PDF) [file pone.0182818.s002.pdf]

Supplementary Table 2. Association of CACNA-1G, CACNA-1H and CACNA-1I with outcome in ovarian cancer

Ovarian cancer								
**Gene**	***n***	**Stage**	**DFS/PFS**			**OS**		
			**HR**	**95%CI**	**p**	**HR**	**95%CI**	**p**
CACNA1G	-/1582	all	not applicable			1.22	1.07-1.40	0.004
	126/133	I-II	1.51	0.80-2.86	0.203	1.55	0.67-3.61	0.305
	494/581	III-IV**	0.84	0.67-1.06	0.145	1.23	0.97-1.57	0.091
CACNA1H	-/1582	all	not applicable			0.82	0.71-0.96	0.011
	126/133	I-II	0.61	0.28-1.32	0.207	0.50	0.20-1.26	0.136
	494/581	III-IV**	0.81	0.63-1.03	0.088	0.64	0.51-0.82	<0.001
CACNA1I	-/1582	all	not applicable			0.84	0.73-0.97	0.018
	126/133	I-II	0.44	0.23-0.82	0.008	0.50	0.17-1.47	0.201
	494/581	III-IV**	1.16	0.90-1.51	0.251	0.64	0.49-0.82	<0.001
Ovarian cancer treated with platinum-containing chemotherapy								
**Gene**	***n***	**Stage**	**DFS/PFS**			**OS**		
			**HR**	**95%CI**	**p**	**HR**	**95%CI**	**p**
CACNA1G	-/1409	all	not applicable			1.24	1.07-1.43	0.004
	94/93	I-II	1.60	0.85-3.04	0.144	1.73	0.66-4.48	0.257
	473/555	III-IV*	0.87	0.70-1.09	0.224	1.27	0.99-1.62	0.059
CACNA1H	-/1409	all	not applicable			0.88	0.75-1.04	0.129
	94/93	I-II	0.58	0.28-1.23	0.151	0.29	0.08-1.01	0.038
	473/555	III-IV*	0.85	0.66-1.09	0.201	0.66	0.52-0.85	<0.001
CACNA1I	-/1409	all	not applicable			0.88	0.75-1.02	0.091
	94/93	I-II	0.44	0.23-0.84	0.010	0.70	0.28-1.79	0.458
	473/555	III-IV*	1.20	0.93-1.55	0.165	0.68	0.53-0.87	0.002

Abbreviations: *n*, number of patients (for first progression [FP] analysis – *i.e.* DFS and PFS – and OS analysis); DFS, disease-free survival; PFS, progression-free survival; OS, overall survival; HR, hazard ratio; 95%CI, 95% confidence interval; p, p-value; *only cases with optimal debulking surgery included.
