# Supplementary figures and images for: Prognostic relevance of a T-type calcium channels gene signature in solid tumours: A correlation ready for clinical validation
Source: PLoS One. 2017 Aug 28;12(8):e0182818. doi: 10.1371/journal.pone.0182818 (PMC5573204; doi:10.1371/journal.pone.0182818)

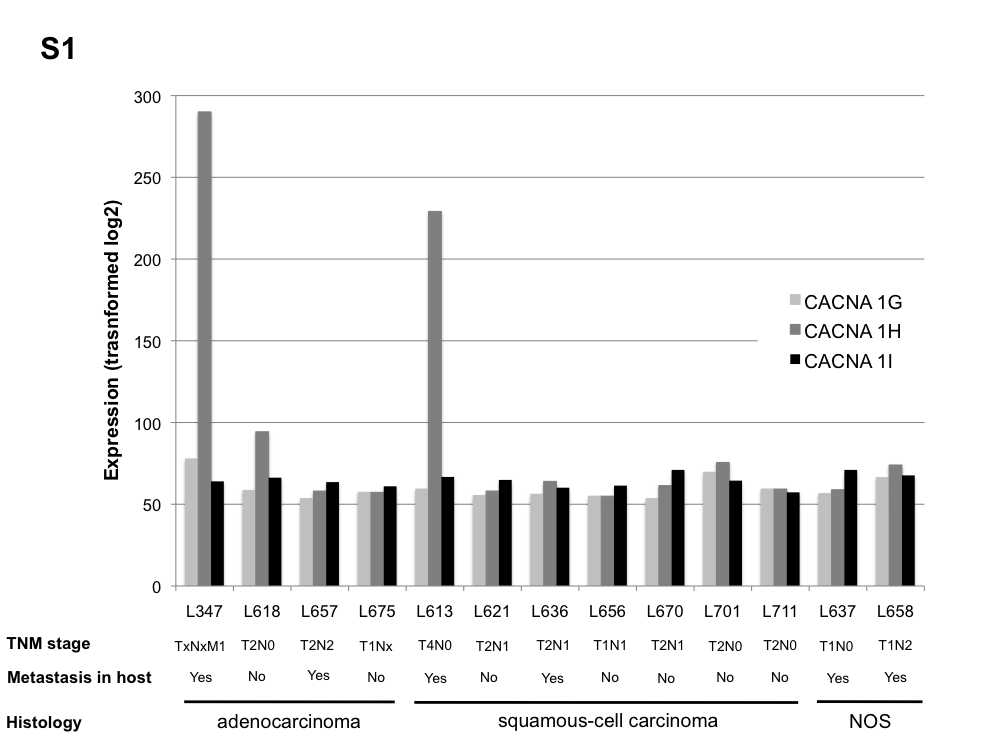

Supplement: S1 Fig — Abbreviations: NOS, not otherwise specified. TNM stage refers to tumour stage in the original patient: all xenografts were derived from primary lung tumours without distant metastases (M0), with the only exception of L347 (derived from a metastatic lung tumour, M1). (TIF) [file pone.0182818.s003.tif]

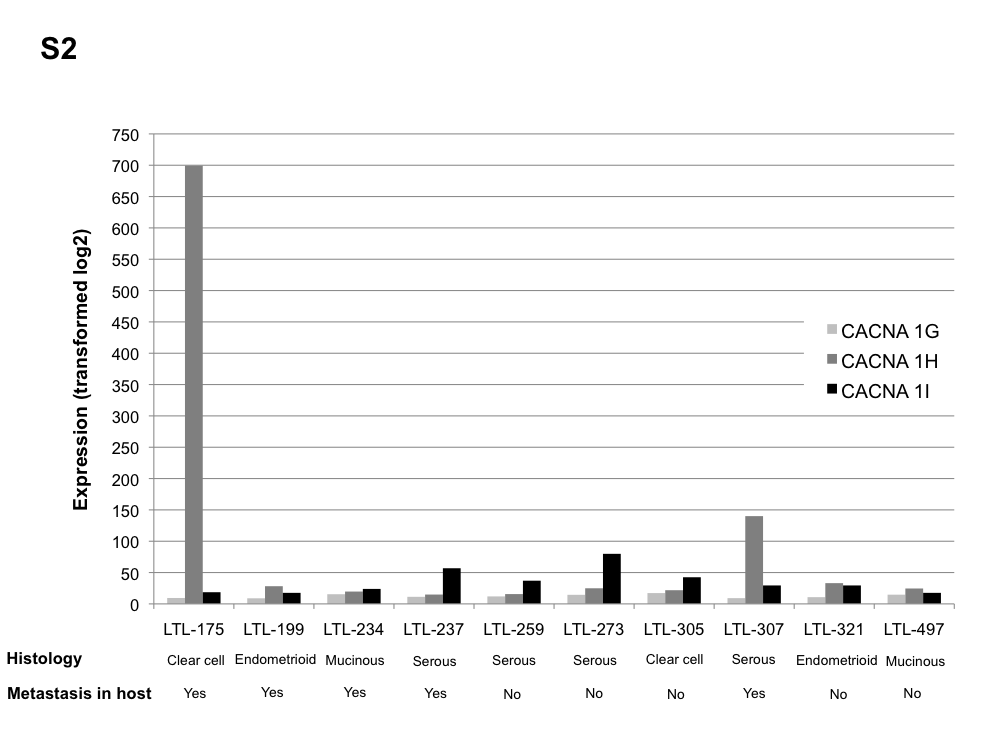

Supplement: S2 Fig — Abbreviations: Histology refers to tumour type in the original patient: all xenografts were derived from primary ovarian tumours. (TIF) [file pone.0182818.s004.tif]

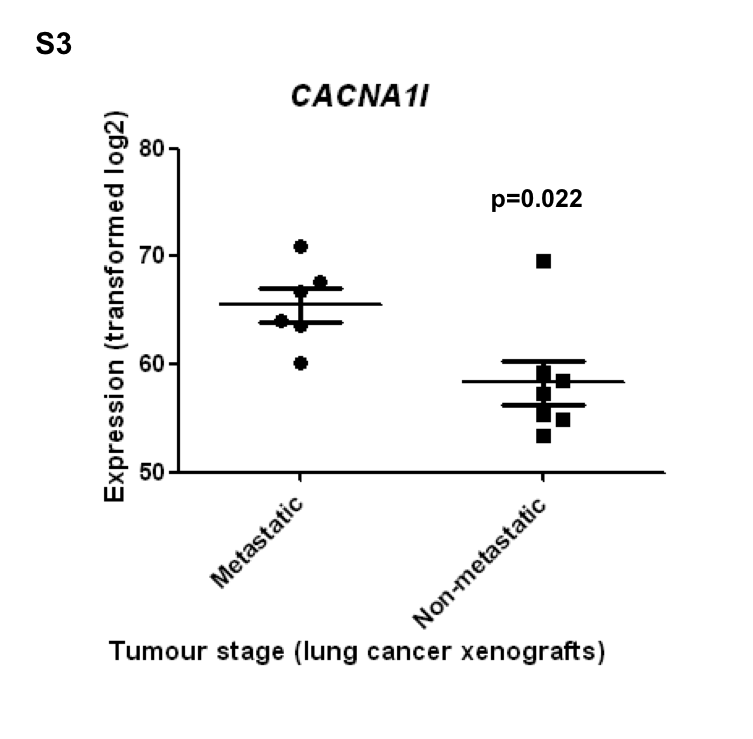

Supplement: S3 Fig — (TIF) [file pone.0182818.s005.tif]
